# Editorial: Efficacy and safety of CAR-T cell therapy in hematologic malignancies

**DOI:** 10.3389/fonc.2024.1472463

**Published:** 2024-08-19

**Authors:** Marcos de Lima, Alice Di Rocco, Jose-Maria Ribera

**Affiliations:** ^1^ Department of Medicine, The Ohio State University, Columbus, OH, United States; ^2^ Department of Translational and Precision Medicine, Sapienza University of Rome, Rome, Italy; ^3^ Department of Clinical Haematology, Hospital Germans Trias i Pujol, Josep Carreras Research Institute, Barcelona, Spain

**Keywords:** chimeric antigen receptor (CAR T), leukemia, myeloma, lymphoma, toxicity

The treatment of CD19- and BCMA-expressing hematologic malignancies has changed dramatically over the last decade since the introduction of autologous chimeric antigen receptor T cells (CAR T) in clinical practice. Although response rates of 20-90% have been observed, only 20-40% of patients with advanced non-Hodgkin’s lymphomas (NHL) and acute lymphocytic leukemia (ALL) are reliably cured with this approach. Disease recurrence remains the major cause of treatment failure in this setting. Furthermore, CAR T cells are associated with specific short- and long-term toxicities, including systemic inflammatory and neurologic syndromes, as well as bone marrow suppression with frequent occurrence of persistent cytopenias. Secondary cancers have also emerged as potential side effect, however with only a small minority having potential direct relationship with insertional mutagenesis.

Approaches to decrease post-CAR T cells relapse rates include utilization of constructs that target more than one antigen. The hypothesis is that this may address antigen escape as a mechanism of relapse (for example, due to decreased expression of CD19 or BCMA), and potentially increase the response rates. Li et al. reviewed 10 studies that reported on the use of anti CD19 and CD22 CAR Ts (or anti CD22 alone) to treat relapsed or refractory B-cell ALL (B-ALL). The pooled complete response (CR) rates were very high, at 0.75 (95% CI: 0.60 - 0.88) and 0.87 (95% CI: 0.76 - 0.96), respectively. The overall negative measurable residual disease (MRD) rates of CD22 and CD19/CD22 CAR-T were 0.54 (95% CI:0.42 - 0.66) and 0.91 (95% CI: 0.47 - 0.88), respectively.

Xu et al. wrote a review on the results of treatment with combined anti-BCMA and anti-CD19 CAR-T cells for relapsed, refractory multiple myeloma. The pooled overall response rate (ORR) was 94% (95% CI: 91%-98%), with a complete response rate (CRR) of 50% (95% CI:29%-71%). The MRD negativity rate of responders was 73% (95% CI: 66%-80%). The pooled all-grade cytokine release syndrome (CRS) rate was 98% (95% CI: 97%-100%), while grade≥3 CRS rate was 9% (95% CI: 4%-14%), and the incidence of neurotoxicity was 8% (95% CI: 4%-11%). Median progression-free survival (PFS) was 12.97 months (95% CI: 6.02-19.91), and the median overall survival (OS) was 26.63 months (95% CI: 8.14-45.11). It is unknown if these results are better than what would be obtained with targeting BCMA alone or if these constructs would offer a salvage strategy for those failing anti-BCMA CAR T cells.

Another strategy to improve disease response is to modify the CAR T cell costimulatory molecules (1). Moreno-Cortes et al. developed and tested a CAR construct with an anti-ROR1 single-chain variable-fragment cassette connected to CD3z by second- and third-generation intracellular signaling domains including 4-1BB, CD28/4-1BB, ICOS/4-1BB, and ICOS/OX40. The authors report that these ROR1.ICOS.OX40z T-cells showed *in vivo* anti-lymphoma activity, a long-lasting central memory phenotype, and improved survival in animal models.

Martino et al. reviewed published data on secondary malignancies after CAR T cells. The authors concluded that ‘caution is needed, both in analyzing reports and communicating their results, to avoid generating unfounded fears to fuel mistrust of therapies now considered lifesaving.’ This statement reflected the fact that several, if not all, cases reported so far are associated with a background of heavily pre-treated diseases, although the incidence of secondary T cell lymphomas, while low, may be higher than expected.

Wang et al. reported on their experience treating 71 patients with relapsed or refractory B-cell ALL or large B-cell lymphoma (LBCL). Data on this cohort was used to develop an early hematotoxicity predictive model. They observed that the incidence was 45.5% and 38.5%, respectively for B-ALL and LBCL, at 3 months after treatment. CRS severity was an independent risk factor for this complex complication. Interestingly, peak cytokine levels of tumor necrosis factor-a (TNF-a) and C-reactive protein (CRP) were closely associated with early hematotoxicity. The authors proposed a predictive algorithm that, if confirmed in larger independent cohorts of patients, will be very useful in identifying patients at risk of post CAR T cells cytopenias.

Cordeiro et al. wrote a comprehensive review of late events using anti-CD19 CAR T cells to treat NHL, defined as those occurring or persisting beyond 1 month after CART infusion. The review focused on cytopenia, immune reconstitution, infections, and subsequent malignancies. Grade 3–4 cytopenia has been reported in 30%–40% of patients and beyond 90 days in 3%–22% of patients. B-cell aplasia and hypogammaglobulinemia (expected on-target off-tumor effects of CART19) have been observed in 44%–53% of patients. Infections beyond 30 days are not frequent and are more common when patients receive subsequent therapies due to disease persistency or progression. Myeloid neoplasm is not rare post-CART, with incidences ranging from 3-8%, but a direct relationship with the CAR transgene has not been found. It is possible that pre-existing mutations and a pro-inflammatory milieu may contribute to this feared development.

Tang et al. reported a case report of a patient with ALL who was treated with autologous CAR T-cell therapy, followed by allogeneic transplant, then another CAR T cell treatment with a donor-derived product. This case reflects some of the questions in the field, such as the use of allogeneic CAR T cells and the potential need to consolidate CAR T responses with allogeneic transplant in some cases of ALL.

Defining and describing toxicities, and subsequently working towards minimizing these adverse events, might contribute to decreasing costs of these therapies, which remain inaccessible to most patients in need due to price. Also, new options to the current centralized manufacturing model are needed, with the goal of increasing access to these life-saving therapies (2, 3). We hope readers will enjoy this article series and benefit from the knowledge summarized here.
